# Reducing Animal Use with a Biotelemetry-Enhanced Murine Model of Sepsis

**DOI:** 10.1038/s41598-017-05497-5

**Published:** 2017-07-26

**Authors:** Anthony Lewis, Brian Zuckerbraun, John Griepentrog, Xianghong Zhang, Matthew Rosengart

**Affiliations:** Department of Surgery, University of Pittsburgh, Pittsburgh, USA

## Abstract

Animal models of sepsis exhibit considerable variability in the temporal development of the physiologic response, which reduces the power of studies, particularly if interventions are tested at arbitrary time points. We developed a biotelemetry-based model of cecal ligation and puncture (CLP) that standardizes the testing of time-sensitive therapies to specific criteria of physiologic deterioration. In this study we seek to further define the variability in physiologic response to CLP sepsis and conduct a cost analysis detailing the potential for reducing animal usage. We have further characterized the variability in physiologic response after CLP in mice and determined peaks in the temporal distribution of points of physiologic decline. Testing therapies at physiologic thresholds reduces the variability found in historical fixed time-based models. Though initial cost is higher with biotelemetry, this is eventually offset by the significantly reduced number of mice needed to conduct physiologically relevant sepsis experiments.

## Introduction

Sepsis remains a clinical problem of immense proportions in both the United States as well as throughout the world^1, 2^. The development of new therapeutics is predicated upon testing in validated animal models, and multiple animal models are available to the modern investigator^3^. A common limitation cited of contemporary animal experimentation is the variability in the host response to septic insult, particularly as it relates to the temporal development of altered physiology^4–7^. The power to investigate the effect of any intervention is inversely related to this variance, and thus, in circumstances of considerable variance, reciprocal increases in sample size are pursued to ensure appropriate power. However, an equally effective means to ensure analytical power is to reduce experimental variability, which carries the additional advantage of reducing animal usage.

Organizations such as the National Centre for Replacement, Refinement, and Reduction of Animals in Research (NC3Rs) have developed guidelines to improve animal welfare in scientific experimentation through the adoption of the “Three Rs”^8, 9^. The ARRIVE guidelines published by this organization have been adopted by over 300 research journals and emphasize minimizing variability, a component that contributes to increased animal use^10^. Specific to the conduct of trials of sepsis, others have proposed that stratification of animal cohorts may facilitate achieving the goals of the “Three Rs” by refining animal models and yielding higher quality experimental data^11^.

While others have stratified experimental animals based upon behavioral or biochemical criteria, our laboratory has developed and validated a biotelemetry animal model that enables quantifying host physiology in real-time, and thereby testing interventions at distinct physiologic endpoints^6, 7, 12–16^. We propose that this method of stratification reduces physiologic variability at the point of testing and is more analogous to human trials of sepsis, which similarly utilize physiologic criteria for subject enrollment^17–20^. We now hypothesize that implementing a biotelemetry-enhanced CLP model will refine murine models of sepsis and reduce animal usage. The data yielded may offer potential utility to investigators in terms of experimental design, reduction of animal use, and allocation of resources.

## Results

### Acute Physiologic Deterioration is Variable after CLP

**Figure 1:**
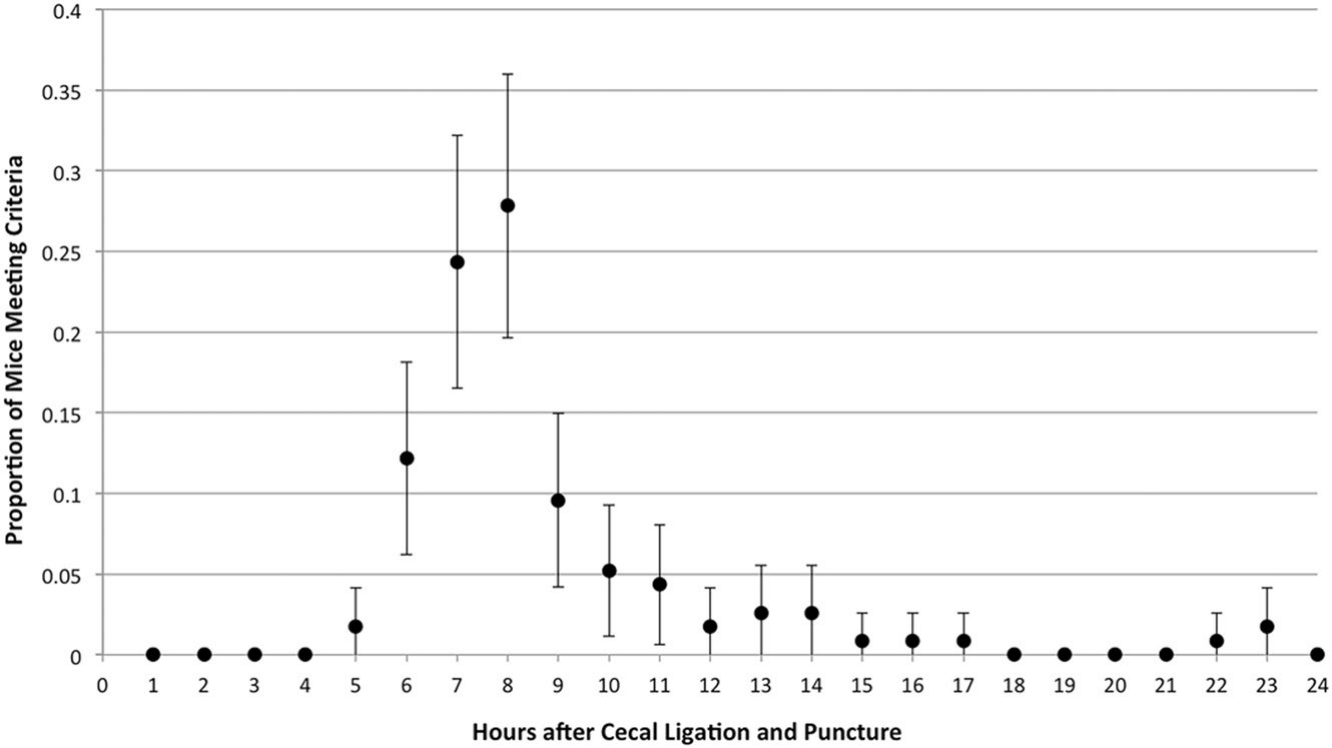
Frequency distribution of mice meeting a threshold of acute physiologic deterioration. The proportion of mice meeting a validated state of acute physiologic deterioration within 30 minutes of each hour after cecal ligation and puncture (n = 115). Data are represented as mean proportion with 95% confidence intervals.

**Figure 2:**
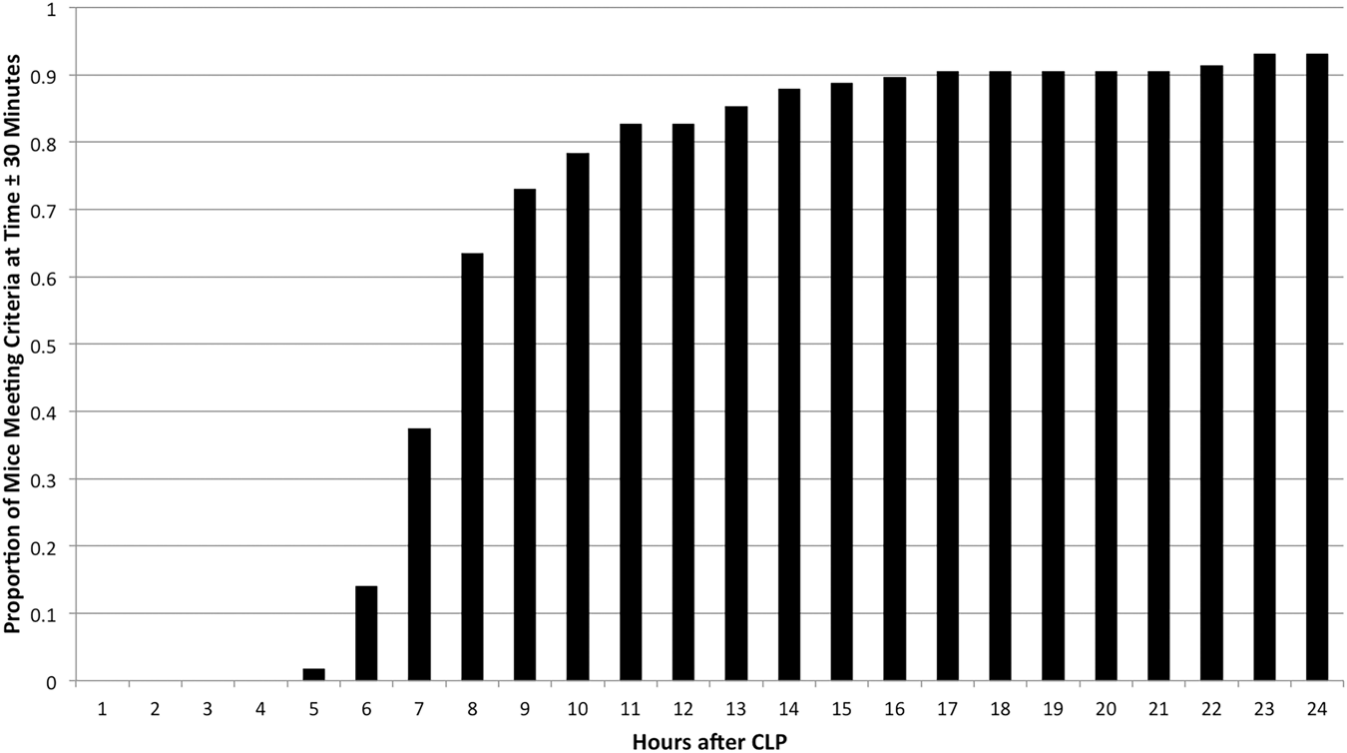
Cumulative proportion of mice meeting threshold for acute physiologic deterioration. The collective proportion of the total cohort (n = 115) of mice which has reached criteria for acute physiologic deterioration within thirty minutes of each hour after cecal ligation and puncture.

Eight of 115 mice (7%) did not meet physiologic deterioration within 24 hours of CLP and were excluded from further analysis. The median time to acute physiologic deterioration (time-to-criteria) was 465 minutes (interquartile range 422–536 minutes, total range 287–1405 minutes) in the remaining (n = 107) cohort. The hourly frequency distribution of time-to-criteria is depicted in Fig. 1. The cumulative frequency is shown in Fig. 2. No single hourly time point after CLP captured a majority (>50%) of mice experiencing physiologic decline. An 8-hour interval after CLP was the single time point possessing the highest proportion of mice meeting the criteria of physiologic deterioration; henceforth referred to as ‘quality mice’ (Table 1). This time point captured significantly more quality mice than 6 or 9 hours after CLP (28% vs. 12%, p = 0.01; 28% vs. 10%, p < 0.01) and though higher than 7 hours, statistically similar (28% vs. 24%, p = 0.70). The 8-hour time point was chosen as a more conservative comparison for subsequent cost analyses. A standard model testing an intervention at this 8 hour fixed time point would include 28% quality mice (95% CI 20–36%), 37% of mice less physiologically deranged, and 36% beyond this severity of physiologic derangement. Using these estimates, a 3.57-fold increase in sample size would be needed to incorporate the same number of quality mice. Thus, for each 120 quality mice tested in a biotelemetry model, a standard CLP model testing at 8 hours would need 429 mice.

**Table 1:** Proportions of mouse at each physiologic state after cecal ligation and puncture.

Hours After CLP	% Meeting Criteria (±30 min.)	Cumulative Proportion at Criteria (±30 min.)	% Mice Past Criteria	% Mice Not Yet at Criteria
4	0%	0%	0%	100%
5	2%	2%	0%	98%
6	12%	14%	2%	86%
7	24%	37%	13%	63%
8	28%	63%	36%	37%
9	10%	73%	63%	27%
10	5%	78%	73%	22%
11	4%	83%	78%	17%
12	2%	83%	81%	17%
13	3%	85%	83%	15%
14	3%	88%	85%	12%
15	1%	89%	88%	11%
16	1%	90%	89%	10%
17	1%	90%	90%	10%
18	0%	90%	90%	10%
19	0%	90%	90%	10%
20	0%	90%	90%	10%
21	0%	90%	90%	10%
22	1%	91%	90%	9%
23	2%	93%	91%	7%
24	0%	93%	93%	7%

Proportions of mice at, beyond, or before criteria for acute physiologic deterioration are presented each hour after cecal ligation and puncture.

### Seasonal Changes Do Not Affect Physiologic Deterioration after CLP

We did not observe significant variation in time-to-criteria by season, despite a small but significantly lower temperature difference in the winter (Table 2). Seven hours (±30 minutes) captured the maximum proportion of quality mice in the spring and fall, rather than the 8 hour time point seen in the pooled cohort of mice (Supplementary Figure S1). There was no correlation between ambient temperature and time-to-criteria (r = 0.08, p = 0.41).

**Table 2:** Seasonal variation in the response to cecal ligation and puncture.

	Winter (n = 29)	Spring (n = 18)	Summer (n = 25)	Fall (n = 35)	*P* Value
Median (IQR) Time to Criteria (minutes)	470 (438–578)	482.5 (427–578)	456 (384–525)	465 (424–511)	0.49
Proportion at Criteria at 8 Hours (±30 minutes)	38%	22%	20%	29%	0.50
Median (IQR) Temperature (Celsius)	20 (19.4–21.1)	22.2 (22.2–22.2)	21.1 (21.1–21.1)	21.1 (21.1–22.2)	<0.001

Sub-cohorts of mice are delineated by season of experimentation. The median time to meeting criteria for acute physiologic deterioration, proportion of mice meeting criteria within the 8 hour time point after cecal ligation and puncture, and median experimental room ambient temperature are calculated for each seasonal cohort of experimental mice.

### Cost Analysis

**Figure 3:**
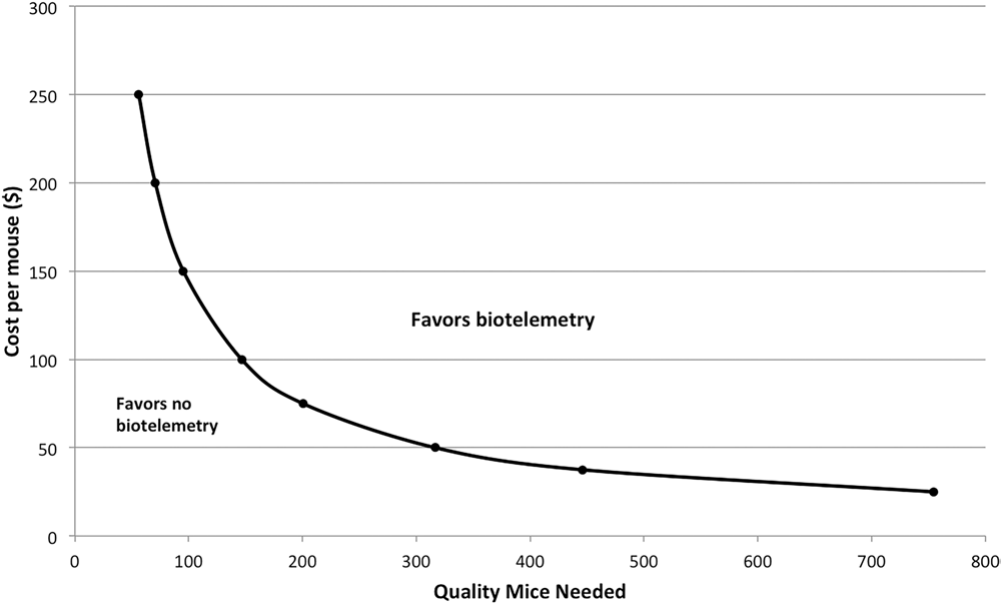
Two-way sensitivity analysis of cost per mouse vs. sample size of quality mice. Sensitivity analysis defines areas of intersection on the plot where model choices can be made purely based upon monetary cost. Area above the curve favors incorporating biotelemetry into cecal ligation and puncture experiments; area below the curve favors cecal ligation and puncture without biotelemetry.

Based upon initial setup costs for 4 new biotelemetry devices and a cost-per-mouse of $25, the incremental cost per quality mouse was $235.27 for the biotelemetry model. The cost-per-mouse spared using biotelemetry was $91.54. A cost-per-mouse of $116.49 rendered the two models equivalent in cost for 120 biotelemetry and 429 8-hour time point mice, each providing 120 quality mice. The cost-per-mouse yielding model cost equivalency diminishes as the number of planned mouse experiments is increased; for example a requirement for 240 quality mice reaches model cost equivalency at a cost-per-mouse of $62.14. Increasing this number to 360 quality mice finds a critical cost-per-mouse of $44.03. One-way sensitivity analyses of total material costs based upon cost per animal and desired number of quality mice is shown in Supplementary Figure S2. The total number of mice spared at model cost equivalency points is summarized in Table 3. For example, at $50 per mouse the models reach cost equivalency at 317 biotelemetry mice (n = 317 quality mice) versus 1130 mice (n = 317 quality mice) without biotelemetry treated at the 8 hour time point, representing a sparing of 813 mice. The incremental cost difference between models decreases in a linear fashion as the cost-per-mouse increases, eventually becoming negative as a biotelemetry-enhanced CLP model becomes more cost-effective (Supplementary Figure S3). A two-way sensitivity analysis of cost-per-mouse versus number of quality mice needed depicts an area above the curve that represents conditions favoring biotelemetry model purely from a cost perspective (Fig. 3).

**Table 3:** Animal quantity at model cost equivalency.

Cost-Per-Mouse (USD)	Biotelemetry Mice	Standard CLP Mice	Mice Spared at Equivalent Cost Point
25	755	2695	1940
37.5	446	1593	1147
50	317	1130	813
75	200	715	515
100	146	523	377
150	95	340	245
200	71	252	181
250	56	200	144

When cost-equivalency is reached, models without biotelemetry use increased numbers of mice to achieve the goal of treating an equivalent sample size of quality mice at acute physiologic deterioration.

## Discussion

Animal studies in the literature, up to this point, have administered experimental treatments at arbitrarily-defined fixed time points after CLP surgery^6, 21–23^. Our study further highlights the variability in the host response to sepsis in one of the most widely applied animal models of sepsis, and that testing at arbitrary time points incorporates a very heterogeneous population. This latter characteristic potentially masks statistical difference through enhanced Type II error, and importantly, detracts from translational relevance to human clinical trials. In response, we developed a more physiologically oriented biotelemetry model and herein, demonstrate its utility in reducing variability, which enhances statistical power and yields considerable reductions in animal usage and ultimately cost. We do propose a time point of 8 hours after CLP in our standard model to capture the highest proportion of C57BL/6J mice within a 30 minute window of acute physiologic deterioration. This information may be valuable for the investigator not currently in possession of or with limited access to biotelemetry technology; it provides a physiologically relevant time point at which therapeutic interventions can be undertaken, albeit at the cost of needing to increase sample size and undertake an initial calibration of the model. Nonetheless, we strongly endorse the further development and utilization of physiologic criteria for the enrollment of animals into the preclinical testing of interventions, so as to more closely mimic enrollment of human subjects in clinical trials.

We previously noted that mice undergoing CLP experienced acute physiologic decline at a wide range of time points after surgery; however this study represents a much larger cohort and more detailed analysis of the exact temporal distributions particularly as it relates to identifying a homogeneous testable septic population^7^. The range of time elapsed until the begin of physiologic decline seen between mice genetically identical in age, weight, and sex is striking, and many factors have been implicated as possible sources of variation in the CLP model^24^. Given the previous reported seasonal variability in sepsis response in both mice and humans, we hypothesized that we would observe similar seasonal differences^25–27^. Although the median time-to-criteria was slightly shorter in the summer and fall, this did not reach statistical significance when compared to winter and spring; and the small temperature changes we observed from day to day in our experiments did not correlate with time-to-criteria. The seasonal differences in murine experimentation observed by Kiank *et al*. may be a reflection of a larger temperature difference between seasons, where a winter to summer difference of 3 °C was noted compared to 1.1 °C in our study^26^. Alternatively, it may represent a response unique to colon ascendens stent peritonitis, a model distinct from CLP; however, we consider this an unlikely etiology^28^. Alterations in light:dark photoperiod length may also contribute, however the constant 12 hour light:dark photoperiod that we used was also used by Kiank *et al*.^26^. Regardless, in the context of making a more clinically relevant mouse model, extrapolating our results to human subjects is confounded by the wide range of temperatures and weather conditions experienced by humans as seasons change. Recreating this wide range of temperatures and weather conditions poses an ethical dilemma in laboratory animals, though may be critically necessary if testing of seasonally specific therapeutics.

By quantifying the hourly proportion of mice attaining deterioration threshold, we identified 8 hours as the best opportunity to treat the most C57BL/6J mice near the point of physiologic decline under our specific laboratory conditions and protocols. However, within an 8 hour ± 30 minute window, over two thirds of mice were at some other physiologic state, either less ill or moribund. Incorporating this physiologic variability may mask the identification of significant differences in treatment effects; indeed a significant minority of mice may never even need treatment. In the past, compensating for this variability in murine models was achieved by increasing the sample size. However, this measure contradicts the goals of the “reduction” arm of the Three Rs^5, 8^. Under the assumptions of our experimental conditions, investigators attempting to overcome model variability by simply increasing sample size would use of over 300 additional mice to ultimately achieve treatment of the same 120 mice at physiologic deterioration.

Without using biotelemetry, it is difficult to assess the exact point of physiologic decline in experimental mice. The manipulation of mice to measure physiologic variables by noninvasive means is confounded by the fear/escape response evoked. Further, without continuous monitoring of physiology, it requires over three times as many mice to achieve the same results in terms of timing of therapeutic treatment, assuming that the overall goal is to deliver treatment to mice at the point of acute physiologic deterioration. This presents a significant opportunity to reduce animal usage by refinement of the CLP model with biotelemetric monitoring of physiology. In addition to defining enrollment points for animal studies of sepsis, it may also be possible to define specific physiologic endpoints for studies as a surrogate for survival analyses, which are frequently criticized as inhumane^11, 14, 29, 30^.

We do acknowledge the increased startup costs with biotelemetry experimentation. However, we further calculated points of cost equivalency based upon cost-per-animal and the number of biotelemetry mice needed for a study. These analyses quantify the mice spared at cost-equivalency points and show potential for considerable reductions in animal usage. Beyond the point of equivalence, animal reduction continues to amass linearly; the incremental cost differences show a steady decline in incremental cost to implement the model as animal costs increase. Though it remains the decision of each lab to determine the worth of such investment, we believe that many groups interested in time-sensitive therapies may find the additional costs worthwhile. Though we used a cost of $25 per mouse as our starting point for cost-per-animal, costs are likely to be much higher, especially with genetically modified or aged mice, or if expensive sample analytic techniques are utilized. We provide a nomogram to facilitate selecting the less costly option depending on the number of biotelemetry mice needed and the cost per mouse. It is important to note that this graph merely guides selection based solely upon cost, ignoring the potentials for animal use reduction, ability to optimally conduct time sensitive studies, and additional physiology data obtained with biotelemetry.

This study is limited by examining only one severity (i.e., 1 cm ligation, 21-gauge double puncture) of CLP, one strain (i.e., C57BL/6J) and sex of mouse, and one laboratory’s protocol. Thus, though appropriate for testing interventions in the context of this particular model, the results do not support rote acceptance of an 8-hour time point of testing; rather they underscore the necessity of defining and addressing host response variability in each and any model. Groups wishing to undertake sepsis experimentation should ideally determine animal pathophysiologic response in their unique environment, taking into account potential genetic strain and sex-linked differences. In reality, there are many ways that the model can vary, and it may be necessary to repeat this analysis in graded CLP severity to calibrate the model and the optimal timing of interventions specific to insult severity^24^. Further, standardizing the severity of insult for supposedly identical CLP models performed by different laboratories could be achieved by comparing the distributions of times to similar points of physiologic deterioration. We are currently limited by testing only one biotelemetry device from a single manufacturer. Other device models and manufacturers exist, and knowledge of the biotelemetry-enhanced CLP concept will be furthered by testing other biotelemetry platforms. Individual differences between systems may be identified and for which accounts may need to be taken, though we believe the overarching concept of the model will remain the same. Finally, it is possible that experimental animals may not exhibit the same peak time to acute physiologic deterioration without monitors implanted; however we have shown previously that there is minimal inflammatory response and rapid mouse recovery after device implantation without CLP^7^. Moreover, the only true way to test this hypothesis without implantable biotelemetry would be to use noninvasive measurements for heart rate and temperature, which require animal restraint, induce a fear/escape response, and artificially alter physiologic parameters.

Biotelemetry-enhanced CLP carries the potential to bring animal models of sepsis to a state more analogous to human clinical trials by incorporating physiologic decline points as enrollment criteria for the testing of interventions. Recent introduction of the Sepsis-3 definition places more emphasis on organ dysfunction as opposed to traditional SIRS criteria in classifying sepsis; however we posit that previous validation of our model offers predictive criterion validity for subsequent development of organ dysfunction and eventual mortality in experimental animals, while avoiding serial invasive sampling^7, 31^. Rather than futile attempts at eliminating variability in the CLP model, continuous physiologic monitoring allows investigators to address this variability in real-time and incorporate it into animal trials. Data from these models can be used to time interventions in experiments not using biotelemetry at the trade-off of the need for larger animal groups. Biotelemetry-enhanced CLP models may substantially decrease the number of mice needed in sepsis experiments, in line with the goals of the “reduction” and “refinement” arms of the Three Rs of ethical animal use in research.

## Methods

### Ethics Statement

All experiments were performed in accordance with the National Institutes of Health guidelines under protocols approved by the Institutional Animal Care and Use Committee of the University of Pittsburgh (Protocol #13021581).

### Study Design

This is a variability and cost analysis of cumulative data obtained in the conduct of CLP sepsis experimentation by our laboratory during a 15-month period from November 2014 to February 2016. We included all mice (n = 115) in this timeframe that underwent a biotelemetry-enhanced model of CLP and had a minimum of 24 hours of physiologic data, regardless of the nature of the original primary experimental hypothesis. This cohort of mice includes mice (n = 27) that were included in our previous publication on biotelemetry-enhanced CLP^7^. However, analysis of time-to-criteria distributions in these mice was not formally undertaken previously. Additional mice (n = 88) were part of other projects within our lab group with various end goals, the common thread being that all mice were treated identically from CLP to the point of acute physiologic deterioration, and no antibiotics were administered during that timeframe.

### Experimental Animals

Male C57BL/6J mice (Jackson Laboratories, Bar Harbor, ME) aged 8 to 12 weeks (mass 25–30 grams) were utilized for all experiments. Mice were housed in specific pathogen-free rooms under 12 hour light/12 hour dark conditions cycled ‘lights on’ at 0700 and ‘lights off’ at 1900. Mice were allowed to acclimate to their surroundings for one week prior to experimentation. Ambient cage temperature (mean ± SD) was 21 °C ± 1 °C. Animals were given *ad libitum* access to water and LabDiet Prolab Isopro RMH 3000 diet pellets (LabDiet, St. Louis, MO). Experiments were conducted in the morning (0700–1000) to control for circadian variation.

### Cecal Ligation and Puncture with Biotelemetry Monitor Implantation

Cecal ligation and puncture (CLP) with concurrent wireless biotelemetry monitor (HD-X11, Data Sciences International, St. Paul, MN) was performed as previously described^7^. Briefly, anesthesia was induced with inhaled isoflurane (2–4%) and intraperitoneal injection of ketamine/xylazine (75 and 6 mg/kg, respectively). A 21-gauge, double-puncture, 1 cm cecal ligation model was used^24, 32^. Monitor implantation into the peritoneal cavity with subcutaneous tunneling of electrocardiogram leads was performed as previously described^7^. At the conclusion of the procedure each mouse received a subcutaneous injection of warmed 0.9% normal saline (30 cc/kg) and was placed on a heating pad until mobile, after which biotelemetry monitoring commenced. All animals received buprenorphine analgesia (0.1 mg/kg SQ every 12 hours). Antibiotics were not administered in an effort to try to recreate the out-of-hospital development of sepsis as closely as possible. Data collection and analysis was performed using Ponemah version 5.20 (Data Sciences International, St. Paul, MN).

Heart rate, core temperature, and animal activity were continuously monitored. We determined the time that elapsed between CLP and acute physiologic deterioration for each mouse^7^. Our physiologic criteria for acute deterioration were previously defined and validated: (1) a 10% decrease in heart rate from peak value, and (2) a decrease in core temperature greater than or equal to 10% of the difference between peak core temperature and 25 °C^7^. This initial portion of the model is the same for each experiment, regardless of the intervention tested or the original primary hypothesis. Mice meeting these physiologic criteria were defined as ‘quality’ mice, insofar that they met physiologic inclusion criteria for enrollment into a preclinical trial.

### Variability Analysis

We calculated the time to physiologic deterioration (i.e., threshold) for each mouse. Mice meeting these physiologic criteria were defined as ‘quality’ mice. Mice that did not meet threshold in the first 24 hours after CLP were excluded from any further analysis. We calculated frequency distributions and cumulative frequencies of mice meeting threshold criteria at each hour (±30 minutes) after CLP, choosing *a priori* to focus on whole integer hour intervals following CLP. We analyzed data from the viewpoint of an investigator performing CLP without biotelemetry, assuming that the goal is to test treatments at the point of acute physiologic deterioration: i.e., on quality mice. Thus, we calculated the hourly time point after CLP that captured the most quality mice experiencing these physiologic parameters within a given tolerance of 30 minutes. Our primary null hypothesis was that no single hour after CLP would capture a majority of animals meeting criteria for acute physiologic deterioration. Conditional on the failure to reject our primary null hypothesis, we additionally tested whether any whole hour time point after CLP was proportionally superior to the others in terms of treating animals at the point of acute physiologic deterioration. Animal and human studies have reported seasonal variation in the septic response, and thus, time-to-criteria data were then further divided and analyzed by season^25–27^. Similarly, the relationship between time-to-criteria and ambient temperature was examined using data recorded in the laboratory logs of room temperature and humidity.

### Cost Analysis

We calculated the incremental costs associated with utilizing the biotelemetry-enhanced CLP model versus a standard time-based CLP model to achieve equal sample sizes of quality mice. Biotelemetry equipment costs were derived from the most recent purchase prices supplied by Data Sciences International (DSI). Each biotelemetry device can perform approximately 30 days of continuous monitoring, or 30 experiments per device with each experiment lasting 24 hours. When battery life has expired, it is possible to have the devices refurbished. Using four devices, a total of 120 single-day monitored mouse experiments can be performed with this setup (which we define as 120 quality mice), and an additional equipment cost is incurred after each 120 biotelemetry mouse interval.

Cost data for the purchase of the biotelemetry equipment including the base hardware, analysis software, and four devices are detailed in Table 4. The cost-per-mouse was the cumulative sum of (1) purchasing the animal from a breeding facility, (2) feeding and housing, (3) routine veterinary care and supervision, (4) pharmacologic agents for anesthesia and analgesia, and (5) experimental sample (e.g., TNFα concentration) analysis. These costs would be expected to vary depending on the nature of experiments being performed (e.g., different mouse strains or analytic techniques), and therefore we examined a range of price-per-mouse costs. An assumption made in cost calculations is the existence of a previously validated biotelemetry-enhanced CLP model. The incremental cost difference was calculated with a numerator consisting of the cost difference between a biotelemetry model and non-biotelemetry model and a denominator of 120 quality mice. The cost of the biotelemetry model included the cost-per-mouse for 120 mice (n = 120 quality mice) and the cost of purchasing monitoring equipment. The cost of the non-biotelemetry model was calculated by multiplying the cost-per-mouse by the number of non-monitored mice needed to capture an equivalent number of quality mice at the point of physiologic deterioration, choosing a fixed time point after CLP surgery for treatment (e.g., 429 mice treated 8 hours after CLP will result in 120 mice treated at the point of acute physiologic deterioration). Sensitivity analyses were performed by varying the number of experimental mice and the cost-per-mouse to identify points of cost equivalency between a biotelemetry and non-biotelemetry model.

**Table 4:** Mean cost estimates.

Equipment	Price Paid (USD)
Monitoring System, Initial Purchase	24336.50
Biotelemetry Devices, Set of Four	11610
Biotelemetry Device Battery Replacement, Set of Four	2405

Cost estimates for equipment utilized.

### Statistical Analysis

Continuous data were compared using either Wilcoxon rank sum or Kruskal-Wallis tests. Correlations of continuous variables were calculated using Spearman’s rank-order correlation. Proportions were compared using Fisher’s Exact Test. Paired dichotomous data were compared using McNemar’s Test. All tests were two-sided. A *P* value < 0.05 was considered significant. Statistical calculations were carried out using Stata 14.1 (StataCorp, College Station, TX).

## Electronic supplementary material

Supplementary Figures
