# Supplementary material for: Reducing Animal Use with a Biotelemetry-Enhanced Murine Model of Sepsis
Source: Sci Rep. 2017 Jul 26;7:6622. doi: 10.1038/s41598-017-05497-5 (PMC5529427; doi:10.1038/s41598-017-05497-5)

Reducing Animal Use with a Biotelemetry-Enhanced Murine Model of Sepsis

Anthony Lewis[1], Brian Zuckerbraun[1], John Griepentrog[1], Xianghong Zhang[1], and Matthew Rosengart[1*]

[1]Department of Surgery, University of Pittsburgh, Pittsburgh, USA

*To whom correspondence should be addressed:
Matthew R. Rosengart, MD MPH
Department of Surgery
200 Lothrop Street
Pittsburgh, PA 15213
412-647-3065
412-647-1448
rosengartmr@upmc.edu

Supplementary Figure S1. Proportion of mice meeting criteria by season. The proportion of mice meeting criteria for acute physiologic deterioration within 30 minutes of each hour after cecal ligation and puncture, calculated by season: (a) winter (n=29), (b) spring (n=18), (c) summer (n=25), and (d) fall (n=35).

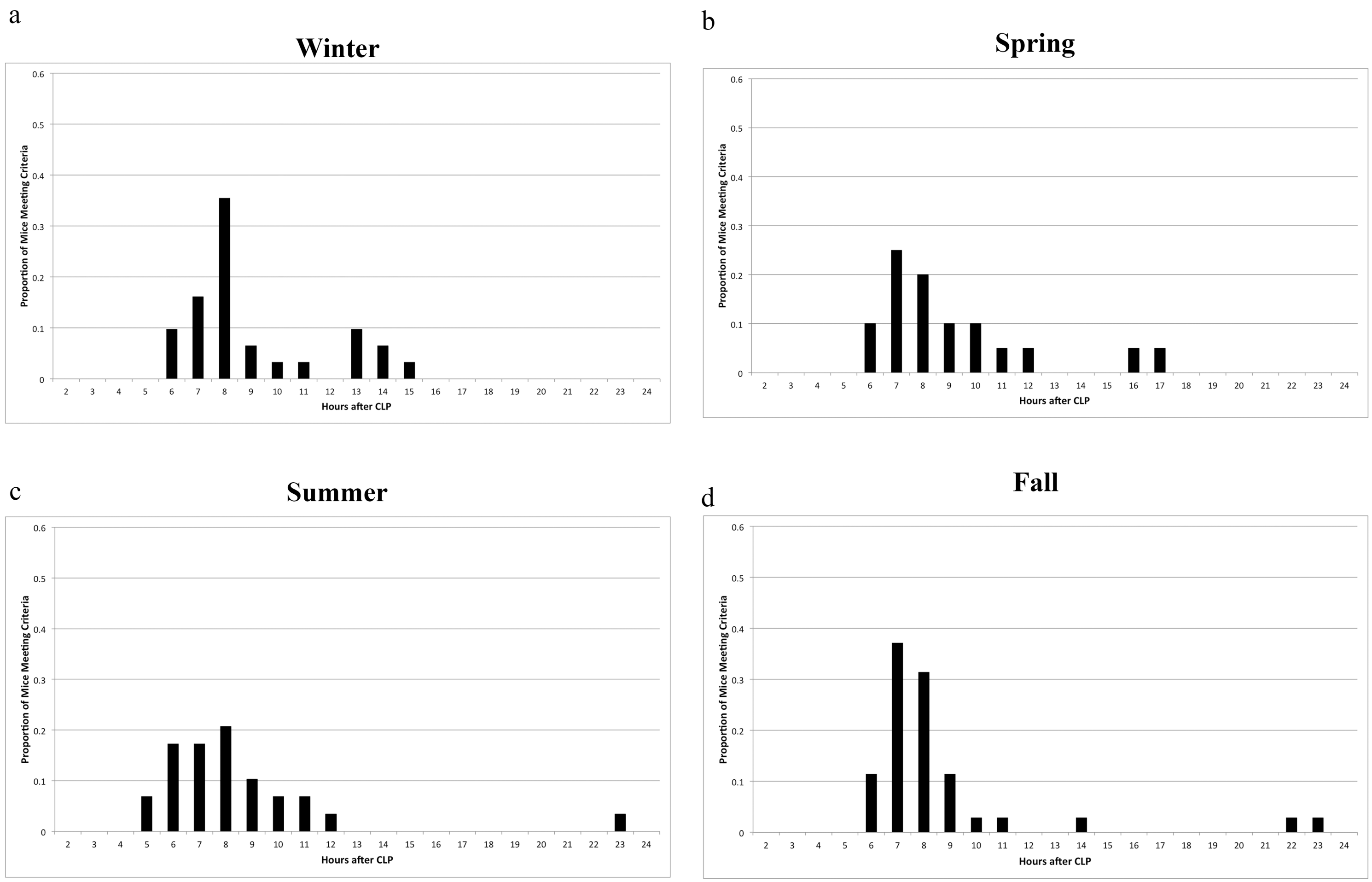

Supplementary Figure. S2. One way sensitivity analyses based upon number of quality mice. Sensitivity analysis plots total material cost for each model as a function of the number of quality mice required for a given experiment. Points of intersection represent cost equivalence. Analyses were conducted for a cost per mouse of (a) $25, (b) $50, (c) $100, and (d) $200.

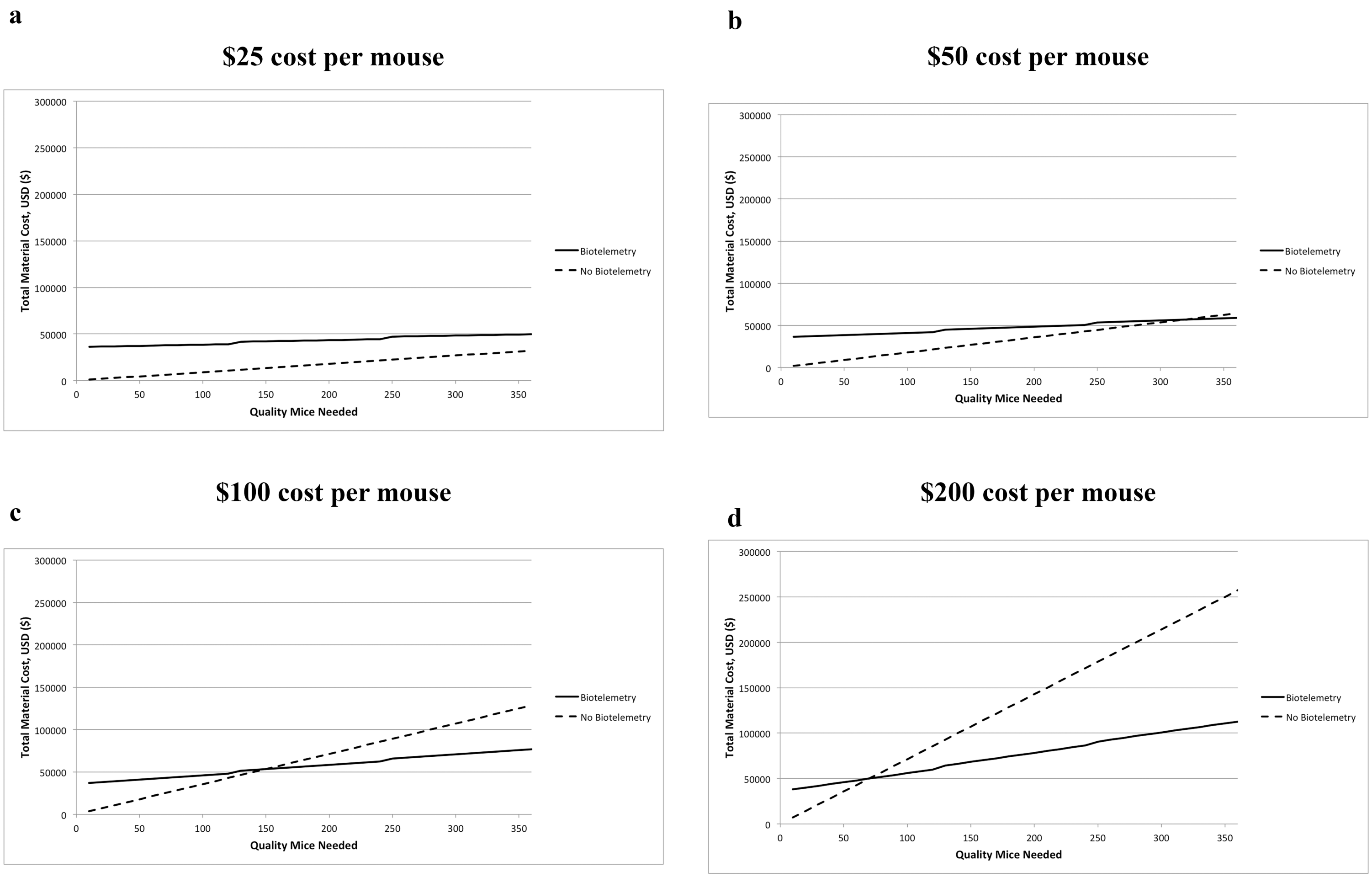

Supplementary Figure. S3. Incremental cost difference for 120 quality mice. Incremental cost differences as a function of increasing cost-per-mouse comparing biotelemetry vs. no biotelemetry models and assuming a sample size of 120 quality mice.

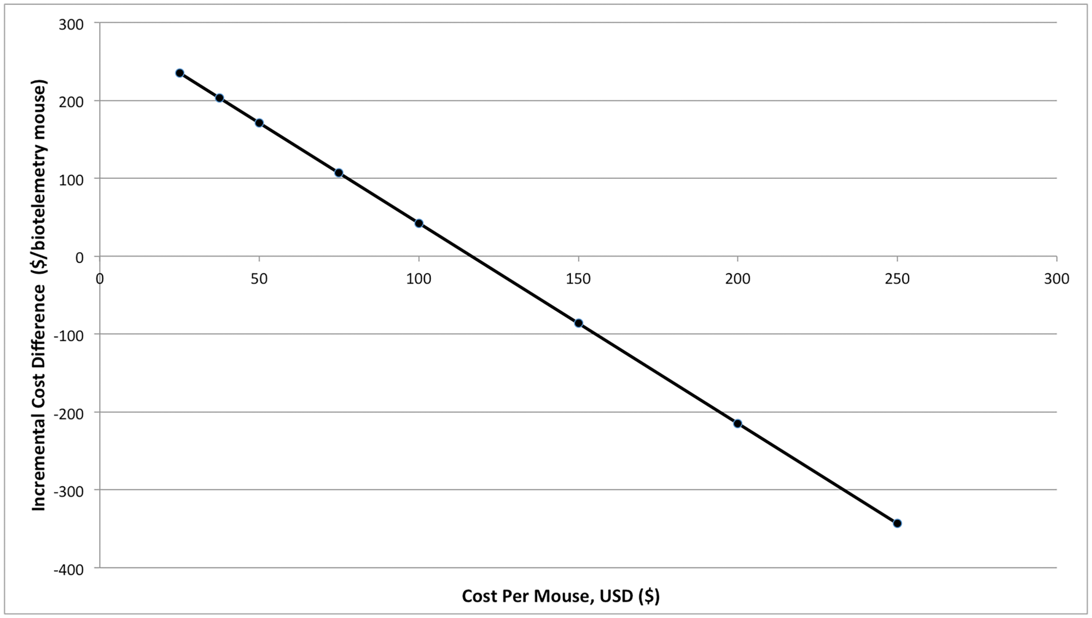

Supplement: Supplementary file 1 — Supplementary Figures [file 41598_2017_5497_MOESM1_ESM.pdf]
